# Magnitude of birth asphyxia and its associated factors among live birth in north Central Ethiopia 2021: an institutional-based cross-sectional study

**DOI:** 10.1186/s12887-022-03500-1

**Published:** 2022-07-18

**Authors:** Nigusie Selomon Tibebu, Tigabu Desie Emiru, Chalie Marew Tiruneh, Bisrat Dessie Getu, Moges Wubneh Abate, Adane Birhanu Nigat, Berihun Bantie, Getasew Legas, Belete Gelaw Walle, Mulualem Gete Feleke, Endalk Birrie

**Affiliations:** 1 Department of Pediatrics and Child Health Nursing, College of Health Sciences, Debre Tabor University, Debre Tabor, Ethiopia; 2 Department of Nursing, Debre Tabor Health Sciences College, Debre Tabor, Ethiopia; 3 Department of Adult Health Nursing, College of Health Sciences, Debre Tabor University, Debre Tabor, Ethiopia; 4 Department of Psychiatry, College of Health Sciences, Debre Tabor University, Debre Tabor, Ethiopia; 5 Department of Pediatrics and Child Health Nursing, College of Health Science and Medicine, Wolaita- Sodo University, Sodo, Ethiopia; 6 Department of Adult Health Nursing, College of Health Science and Medicine, Wolaita- Sodo University, Sodo, Ethiopia; 7 Department of Pediatrics and Child Health Nursing, College of Medicine and Health Science, Wollo University, Dessie, Ethiopia

**Keywords:** Birth asphyxia, Live birth, Hospital, Ethiopia

## Abstract

**Background:**

The leading cause of neonatal death worldwide is birth asphyxia. Yearly, in the first month of life, 2.5 million children died around the world. Birth asphyxia is a major problem, particularly in developing nations like Ethiopia. The goal of this study was to determine the magnitude of birth asphyxia and the factors that contributed to it among neonates delivered at the Aykel Primary Hospital in north-central Ethiopia.

**Methods:**

From August 1 to August 31, 2021, a hospital-based cross-sectional study was conducted on 144 live births. An Apgar score less than 7 in the fifth minute of birth authorized the diagnosis of birth asphyxia. Variable contention (*P* < 0.250) for multivariable analysis was determined after data examination and cleaning. Then, to identify important factors of birth asphyxia, a multivariable logistic regression model with a *p*-value of 0.05 was developed. Finally, a significant relationship between a dependent variable and independent factors was defined as a *p*-value less than 0.05 with a 95% confidence interval.

**Results:**

The majority of the mothers, 71.53%, received at least one Antenatal care visit, and more than half of the newborns were male (62.50%). The percentage of neonates that had asphyxia at delivery was 11.11% (95% CI: 6.3 -16.9%). Male newborns were 5.02 times more probable than female newborns to asphyxiate [AOR: 5.02, 95% CI (1.11–22.61)]. Mothers who have not had at least one Antenatal Care visit were 3.72 times more likely to have an asphyxiated newborn than those who have at least one Antenatal Care visit [AOR: 3.72, 95%CI (1.11–12.42)]. Similarly, mothers who had an adverse pregnancy outcome were 7.03 times more likely to have an asphyxiated newborn than mothers who had no such history [AOR: 7.03, 95% CI (2.17–22.70)].

**Conclusion:**

Birth asphyxia in newborn has come to a standstill as a major public health issue. The sexual identity of the newborn, Antenatal Care visits, and a history of poor pregnancy outcomes were all found to be significant risk factors for birth asphyxia. These findings have great importance for various stakeholders who are responsible for reducing birth asphyxia; in addition, policymakers should establish and revise guidelines associated to newborn activities and workshops.

## Background

The World Health Organization (WHO) defines birth asphyxia as the failure to initiate and maintain breathing after birth [1].

Birth asphyxia occurs when the fetus receives insufficient oxygen, usually during labor and childbirth, resulting in the risk of death (stillbirth or neonatal death) or lifelong disability in the existing infant, and cognitive difficulties can also be predicted due to the pattern of brain injury associated with neonatal encephalopathy [2, 3].

When a newborn has an Apgar score less than seven, it is diagnosed as having birth asphyxia. Scores of four to seven indicate moderate birth asphyxia, while zero to three indicates severe birth asphyxia [4, 5].

Worldwide and yearly, 2.5 million children died in the first month of life which added 47% of deaths of under the age of 5-years and 54% of all under-five deaths occur during the neonatal period among African newborns [6, 7]. In addition, maternal care, such as perinatal care (example Antenatal Care (ANC), has declined, resulting in maternal and newborn health issues, as the article states, one-third had less than one visit per trimester [8].

The neonatal death rate in Ethiopia is 30 per 1000 live births. With 47/1000 live births, the Amhara region has the highest rate of newborn mortality [9]. The findings demonstrate that birth asphyxia is a widespread public health issue with varying levels of importance across the country [9, 10]. For example, birth asphyxia is accountable for 31.6% of Ethiopian newborn death [11].

As a solution, Ethiopia has developed a variety of initiatives, including expanding ANC coverage, skilled birth attendance, and postnatal care [12]. Because there is no recent data that reveals the magnitude of birth asphyxia in primary hospitals, this study has the ability to provide observed data about birth asphyxia. It is intended to fill the gap and contribute to picking up the magnitude of birth asphyxia in primary hospitals. Therefore, this study was aimed to determine the magnitude and associated factors of birth asphyxia among newborns admitted at Aykel primary hospital in North West Ethiopia.

## Methods and materials

### Study design and study period

A hospital-based cross-sectional study was conducted from August 1 2021 to August 31, 2021.

### Study area

The research was carried out at the Aykel basic hospital in Aykel town, which was founded in 2002 and is located 802 km from Addis Ababa, Ethiopia’s capital city. The hospital includes diverse units; the maternity unit, which has 35 beds, handles more than 3000 clients every year.

### Source population and study population

Source Population

- All live birth neonates gestational age 28 weeks or more in Aykel primary hospital

Study Population

- All live birth neonate’s gestational age 28 weeks or more at Aykel primary hospital during data collection time.

Exclusion Criteria

- Newborns of unknown gestational age at birth, and newborns with malformations

### Sample size determination

The sample size was determined by using a single population proportion formula with a 95% confidence level and 5% margin error. The actual sample size for the study was computed by using $\mathrm{n}=\frac{\mathrm{p}\left(1-\mathrm{p}\right){\mathrm{z}}^2}{{\mathrm{w}}^2}$, Where; Z = is the confidence level at a certain value of significance, *n* = sample size calculated, Z = level of significance at 95% of confidence interval = 1.96, P = proportion of birth asphyxia from the similar study which was done from Debre Tabor General Hospital (28.35%) [13], w = marginal of error 5% (0.05 then $\mathrm{n}=\frac{0.2835\left(1-0.2835\right){(1.96)}^2}{(0.05)^2}=312$, after adding 10% for non-response the final sample size was144.

Sampling technique and procedure

- All Mothers who gave live birth after 28 weeks of gestational age, and had informed consents were incorporated for this study.

### Study variable

Dependent variable

- The magnitude of birth asphyxia

Independent variable

- Socio-demographic variables (age, educational level, residence, occupation)
- Maternal Characteristics (ANC visit, Sex of neonate, birth weight, Birth type, history of adverse pregnancy outcome, obstetrics complication during pregnancy, time of delivery)

### Operational definition

**Birth asphyxia:** A newborn was considered to have birth asphyxia when its fifth minute Apgar score was < 7 [14, 15].

**Preterm:** is birth of the baby before 37 completed weeks of gestational age.

**Post-term**: is birth of the baby after 42 completed weeks of gestational age.

**Obstetric complication**: Mal presentation, malposition, prolonged labour or obstructed labour, or/and others [16].

**Previous adverse pregnancy outcome:** Yes, were considered when a pregnancy ends with at least one of unwanted pregnancy outcomes.

### Data collection tool and procedure

Data was collected using pretested and structured questionnaires. Interview was done after delivery and when the mother was stable. Primary data on socio-demographic variables and maternal characteristics with birth-related factors was collected using an interviewer-administered questionnaire. Because twin neonates share a similar sociodemographic background and antenatal history, each mother was only questioned once about these characteristics for twin deliveries.

Reviewing records revealed the Apgar score for the fifth minute (charts). Diploma nurses obtained the data. Nurses with master’s degrees were in control of the supervision. Data collectors and supervisors received one-day training prior to data collecting.

### Data quality control

For data collection, the questionnaire was originally written in English. However, it was translated into Amharic and then back into English to ensure uniformity. Pre-testing the question on 5% of the sample in the research area before the actual data collection period for 3 weeks improved the data quality. The necessary adjustments were made after the pretest. Supervisors double-checked the data to make sure it was accurate.

### Data processing and analysis

The data was carefully reviewed for correctness before being coded and entered into SPSS version 20. Variable candidacy (*P* < 0.250) in binary for multivariable analysis was determined after data exploration and cleaning. Then, to identify important factors of birth asphyxia, a multivariable logistic regression model with a *p*-value of 0.05 was developed. Finally, a significant relationship between a dependent variable and independent factors was defined as a *p*-value less than 0.05 with a 95% confidence interval.

## Result

### Socio-demographic, maternal, and newborn related characteristics

With reviewed charts, there were a total of 144 mother-newborn pairs. 65 (45.13%) of the total participants had a secondary education or more, and nearly half (47.91%) of the mothers were between the ages of 26 and 35. The majority of mothers, 71.53%, had at least one antenatal care visit, and nearly half of the babies were male (62.50%) (Table 1).

**Table 1:** Characteristics of Participants for Birth asphyxia among Live birth at Aykel Primary Hospital North Central Ethiopia 2021

Characteristics	Categories	Frequency(N)	Percentage (%)
Mothers age group	15–25 years	56	38.9
	26–35 years	69	47.91
	36–45 years	19	13.19
Mothers’ education level	Unable to read and write	22	15.29
	Primary education	24	16.67
	Secondary and above	65	45.13
	College and above	33	22.91
Gestational age	Preterm	17	11.80
	Term	110	76.38
	Post-term	17	11.80
Birth type	Single	134	93.06
	Twin	10	6.94
Birth weight	1–1.5 kg	6	4.17
	1.5–2.5 kg	2	1.39
	2.5–4.0 kg	130	90.27
	4+ kg	6	4.17
Time of delivery	Night	68	24.00
	Day	76	76.00
Sex of neonate	Male	90	62.50
	Female	54	37.50
ANC visits	Yes	103	71.53
	No	41	28.47
History of adverse pregnancy outcome	Yes	31	21.53
	No	113	78.47
Obstetrics complication during pregnancy	Yes	36	25.00
	No	108	75.00
Obstructive complication (*N* = 36)	Preenclampsia/enclampsia	13	36.10
	Ante partum hemorrhage	4	11.11
	Anemia	11	30.56
	Infections	6	16.67
	Gestational diabetes	2	5.56
Residence	Rural	68	47.23
	Urban	76	52.77
Occupation	House wife	97	67.36
	Governmental employee	41	28.47
	Merchant	4	2.78
	Daily laborer	2	1.39

### Prevalence of birth asphyxia

The overall magnitude of birth asphyxia among live birth was 11.11%(95% CI: 6.3–16.9).

### Factors associated with birth asphyxia among newborn in Aykel primary hospital Amhara region of Ethiopia

With a *p*-value of 0.250, the bivariable analysis revealed that the neonate’s sex, delivery time, ANC visits, history of bad pregnancy outcome, and obstetrics complication during pregnancy were all related to birth asphyxia. The factors that were significant in bivariable analysis were entered into multivariable logistic regression to adjust possible confounders. The findings revealed that the neonate’s sex, ANC visits, and a history of adverse pregnancy outcomes were all linked to delivery hypoxia. Male newborns were 5.02 times more likely than female newborns to become asphyxiated [AOR: 5.02, 95% Confidence interval (1.11–22.61)]. Mothers who had not have at least one ANC visit were 3.72 times more likely than those who had at least one ANC visit to have an asphyxiated newborn [AOR: 3.72, 95% CI (1.11–12.42)]. Similarly, mothers with a history of adverse pregnancy outcomes were 7.03 times more likely to have an asphyxiated newborn than those without a history [AOR: 7.03, 95% CI (2.17–22.70)] (Table 2).

**Table 2:** Bivariate and Multivariable Logistic Regression Analyses for Birth asphyxia among Live birth at Aykel Primary Hospital North Central Ethiopia 2021

Variables	Categories	Total *N* = 144	Asphyxia		COR/95% CI/	AOR/95% CI/
			Yes	No		
Sex of neonate	Male	90(62.5%)	13	77	2.87 (0.78–10.56)	5.02(1.11–22.61)*
	Female	54(37.5%)	3	51	1	1
Time of delivery	Night	68 (47.2%)	10	58	2.01(0.69–5.87)	0.95(0.23–3.91)
	Day	76 (52.8%)	6	70	1	1
ANC visits	Yes	103(71.5%)	8	95	1	1
	No	41(28.5%)	8	33	2.88(1.00–8.28)	3.72(1.11–12.42)*
History of adverse pregnancy out-come	Yes	31(21.6%)	9	22	0.16(0.54–0.48)	7.03 (2.17–22.70)**
	No	113(78.4%)	7	106	1	1
Obstetrics complication during pregnancy	Yes	36(25.0%)	9	27	4.81(1.64–14.09)	3.09 (0.77–12.29)
	No	108(75.0%)	7	101	1	1

Notes: 1 = reference group, *significant *p*-value< 0.05, **significant *p*-value< 0.01, *CI* confidence interval; *COR* crude odds ratio; *AOR* adjusted odds ratio

## Discussion

The goal of this study was to find out what factors might be linked to birth asphyxia. The findings demonstrated that the neonate’s sex, ANC visits, and a history of adverse pregnancy outcomes were all associated to birth asphyxia.

The overall magnitude of birth asphyxia in this study was 11.11% (95% CI: 6.3–16.9). When compared to a research conducted in Dira Dawa, Ethiopia (3.1%), it is higher [17]. The possible reason might be due to the period of the study, and existence of health services. However, It is lower than a study done at Amhara referral hospital Ethiopia (22.6%) [4] Debre Tabor General hospital Ethiopia (28.35%) [13], Debre tabor (29.9%) [18], Wolita Sodo, Ethiopia (27.5%) [19], overall in Ethiopia (19.3%) [16], Gusua Hospital Nigerai (30.1%) [20].

The possible reasons might be due to the application of updated guidelines, and most studies were referral hospitals while this study was particular to one primary hospital, and this leads to increase number of existence of cases typically birth asphyxia.

On the other hand, this study was in line with the study done Jimma Public Hospital Ethiopia (12.5%) [14], Nigist Eleni Mohammed memorial teaching hospital, Southern Ethiopia(15.1%) [21].

The magnitude and determinants of birth asphyxia among live newborns at Ayikel Primary Hospital are presented in this report. Birth asphyxia was found to be significantly predicted by sex of the neonate, ANC visit, History of adverse pregnancy outcome.

The sex of the newborn was found to be one of the predictors of birth asphyxia in this investigation. A male newborn was 5 times more likely than a female newborn to become asphyxiated. This finding is supported by a study done in sexually dimorphic outcomes [22, 23], and the finding concluded as possibility of striking innate immunological response that is sex-specific. Beside to this, researchers can investigate further, the depth of the relationship between baby sexual identification and birth asphyxia.

A mother ANC visit was also a predictor of birth hypoxia. Mothers who did not have at least one ANC visit were 3 times more likely than those who did have at least one ANC visit to have an asphyxiated newborn. A study conducted in Ethiopia systematic review and Meta analysis, Civil Hospital Dow University, Surin Hospital, Soetomo Hospital, and Pattani Hospital Thailand [16, 24–27] supports this conclusion respectably. This resemblance might be caused by the fact that ANC frequently use results in an early identification of complications, which was a significant component in this investigation. Because the current antenatal care model, which primarily addresses medical hazards, needs to change in order to encompass non-medical elements and encompass the entire community, and services must be multidisciplinary in order to treat both social and medical difficulties. Not only should clinics be equipped to handle complex obstetrical concerns, but also social, mental, and addiction issues as well as chronic diseases like obesity, hypertension, diabetes, and infections [28].

In underdeveloped nations, a history of adverse pregnancy outcomes has a significant impact on the occurrence of birth asphyxia for live birth. In this study, mothers who had a history of bad pregnancy outcomes were 7 times more likely to have an asphyxiated baby than mothers who had never had a adverse pregnancy out-comes. Furthermore, this study is supported by a study done in Jimma Public Hospital, Civil Hospital Dow University, Soetomo Hospital, Pattani Hospital Thailand, and University of Gondar Hospital [14, 24, 26, 27, 29] respectively. This might due to that history of adverse pregnancy outcomes may be ensured by physical or psychological impact on the mother, and this may be lead to an adverse out come on the current pregnancy [30].

### Limitation of the study

The study was conducted with a cross-sectional study design, thus it does not prove a cause and effect relationship, and the study was done only live birth which doesn’t include all newborns. In addition, the data were collected within a short period which couldn’t cover annual served activities. To diagnose asphyxia, we evaluated the Apgar score and clinical symptoms. Our Apgar scores are thought to have been issued appropriately by the maternity personnel on hand at the time of delivery. However, using the Apgar score alone to diagnose and predict the result of birth asphyxia has been proved to be unreliable.

## Conclusion

Birth asphyxia in neonates has come to a standstill as a major public health issue. The sexual identity of the newborn, ANC visits, and a history of poor pregnancy outcomes were all found to be significant risk factors for birth asphyxia. These findings have significant importance for various stakeholders who are responsible for reducing birth asphyxia; in addition, policymakers should establish and revise guidelines associated to newborn activities and workshops. Moreover, researchers use other study design (like survival of asphyxiated newborn) to recognized left out variables like survival, admission to neonatal unit, need for breathing support, seizures and encephalopathy.

## Data Availability

The datasets used and/or analyzed during the current study are available from the corresponding author on reasonable request.

## Abbreviations

ANC: Antenatal Care
Apgar: Appearances Pulse Grimace Activity Respiratory
SPSS: Statistical Package for Social Science
WHO: World Heal Organization
